# Cohort profile: The Multiethnic Lifestyle, Obesity and Diabetes Registry in Malaysia (MeLODY) retrospective cohort in a middle-income country in Southeast Asia

**DOI:** 10.1371/journal.pone.0331571

**Published:** 2025-09-09

**Authors:** Sarkaaj Singh, Anis Syazwani Abd Raof, Jian-Wen Samuel Lee-Boey, Hana Salwani Mohd Zaini, Ying Guat Ooi, Lee-Ling Lim

**Affiliations:** 1 Department of Medicine, Faculty of Medicine, Universiti Malaya, Kuala Lumpur, Malaysia; 2 Information Technology Department, Universiti Malaya Medical Centre, Kuala Lumpur, Malaysia; 3 Department of Medicine, Universiti Malaya Medical Centre, Kuala Lumpur, Malaysia; 4 Department of Medicine and Therapeutics, The Chinese University of Hong Kong, Hong Kong SAR, China; 5 Asia Diabetes Foundation, Hong Kong SAR, China; 6 Baker Heart and Diabetes Institute, Melbourne, Victoria, Australia; Universiti Tunku Abdul Rahman Fakulti Perubatan dan Sains Kesihatan M Kandiah, MALAYSIA

## Abstract

There is a lack of longitudinal data on type 2 diabetes (T2D) in low- and middle-income countries. We leveraged the electronic health records (EHR) system of a publicly funded academic institution to establish a retrospective cohort with longitudinal data to facilitate benchmarking, surveillance, and resource planning of a multi-ethnic T2D population in Malaysia. This cohort included 15,702 adults aged ≥ 18 years with T2D who received outpatient care (January 2002-December 2020) from Universiti Malaya Medical Centre (UMMC), Kuala Lumpur, Malaysia. The mean age of participants was 54.3 ± 12.6 years, with a T2D duration of 12.7 ± 4.8 years, HbA_1c_ of 8.9 ± 2.6%, body mass index of 28.2 ± 6.2 kg/m^2^, and 47.4% were men. The top three comorbidities were dyslipidaemia (87.1%), overweight/obesity (69.4%), and hypertension (62.6%). The proportion of participants achieving HbA_1c_ < 7%, blood pressure < 130/80 mmHg, and low-density lipoprotein cholesterol < 2.6 mmol/L was 27.8%, 24.8%, and 24.5%, respectively. The most common treatments were metformin (62.4%), sulfonylurea (32.8%), and insulin (32.7%). Given the lack of implementation of urinary albumin:creatinine ratio for early detection, chronic kidney disease (defined as estimated glomerular filtration rate < 60 mL/min/1.73m^2^) was underestimated at 7.5%. These findings highlight opportunities for improved data collection in a middle-income country in Southeast Asia. Apart from trend analysis, this cohort will be prospectively followed for ongoing benchmarking, surveillance, and ascertainment of clinical events, including death.

## Introduction

Malaysia is an upper-middle-income country with a diverse population of 34.3 million and a gross domestic product (GDP) per capita of USD 11,648 in 2023 [1]. The population consists of three major ethnic groups (52.3% Malays, 20.1% Chinese, and 5.9% Indians) [2]. Malaysia has a dual-track healthcare system, with 52.3% of healthcare expenditure being borne by the government [3]. In 2024, non-communicable diseases (NCDs) in Malaysia were projected to impose an economic burden of RM 64.2 billion (USD 14 billion), equivalent to 4.2% of the nation’s GDP [4]. This substantial cost is driven primarily by indirect costs, particularly vision loss and premature deaths, with NCDs accounting for 72% of such cases [4–6].

Among these, diabetes stands out as a significant concern, posing a substantial challenge to the healthcare system in Malaysia. Based on the 2023 Malaysian National Health and Morbidity Survey, the prevalence of adults with diabetes climbed from 11.2% in 2011 to 15.6% in 2023, corresponding to a total of 3.6 million people [7]. Indians had the greatest prevalence of type 2 diabetes (T2D) at 26.4%, followed by Bumiputera Sarawak (17.2%), Malays (16.2%), and Chinese (15.1%) [8]. People with diabetes in Malaysia frequently exhibited suboptimal adherence with dietary recommendations, consumed high amounts of carbohydrates, and led sedentary lifestyles [9]. Among people with diabetes who sought treatment under publicly funded healthcare facilities, 70% of them were managed by primary care clinics, imposing a substantial burden owing to limited staffing and resources [9]. Diabetes, hypertension, obesity, and dyslipidaemia are major risk factors for cardiovascular disease. These cardiometabolic risk factors often coexist, complicating the management and outcome [7].

Data is crucial for identifying gaps in T2D care, guiding policy decisions, and implementing evidence-based interventions to improve health outcomes [10]. Currently, there is a lack of integrated electronic health record (EHR) systems in Malaysia for benchmarking and surveillance. The Malaysian National Diabetes Registry (NDR), established in 2009, only included people with T2D who received care at primary care clinics managed by the Ministry of Health [11]. The NDR excluded those who received long-term care at hospitals with different risk profiles, comorbidity patterns, and treatment availability. Herein, by leveraging the EHR system of a publicly funded academic institution, we aimed to establish a retrospective cohort with longitudinal data to facilitate benchmarking, surveillance, and resource planning of a multi-ethnic T2D population in Malaysia.

## Methods

### Setting

The Universiti Malaya Medical Centre (UMMC) is a publicly funded academic institution with 1,681 beds [12]. Located in the capital city of Kuala Lumpur, UMMC offers a comprehensive range of medical services, including outpatient primary care, an integrated diabetes centre, and numerous subspeciality services. The EHR system at UMMC was initiated in 2012, undergoing a stepwise transition from manual documentation to a fully electronic platform. Diagnosis coding (ICD-10), procedure coding, and the electronic prescribing system were introduced stepwise since 2013.

### Data collection

**Fig 1:**
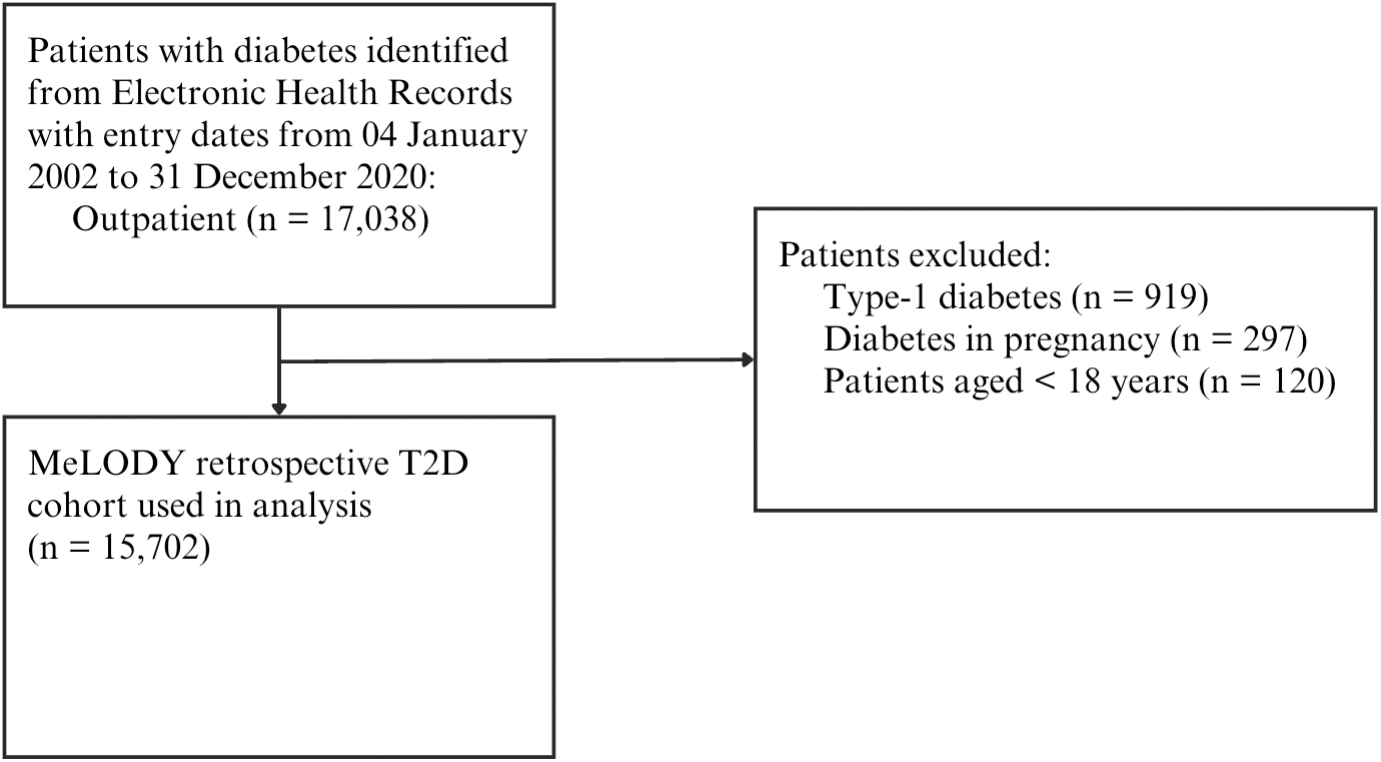
MeLODY Study flow diagram for the setting up of the MeLODY retrospective T2D cohort.

People aged ≥ 18 years with T2D who received outpatient care in UMMC from 4 January 2002 to 31 December 2020 were identified from the EHR, with data accessed on 31 March 2021. Baseline demographics, anthropometric and vital signs, comorbidities, laboratory results, and pharmacy prescription data were extracted from the EHR. In high-income countries, coding administrative assistants are commonly employed to ensure coding accuracy and improve time efficiency [13]. However, in many low- and middle-income countries (LMICs), including Malaysia, such resources are often unavailable, placing the burden of coding on physicians. This leads to variability and inconsistency in coding practices. Therefore, a text-mining algorithm was developed to identify comorbidities listed in EHR entries by physicians. Validation techniques demonstrated that the combined algorithm and manual rectification process achieved a sensitivity of 99.5%, a specificity of 96.7%, and a positive predictive value of 99.4%. All comorbidities identified were re-coded using the ICD-10 code. Diagnostic data were extracted from both outpatient and inpatient medical records to ensure completeness and accuracy of comorbidity identification [14]. The duration of diabetes was estimated using a machine learning model (S1 Text) trained on a cohort with a high degree of similarity in Malaysia [15]. Fig 1 summarizes the process of the cohort selection.

### Sample size

As this study used routinely collected data from the UMMC EHR, the availability of data across domains varied by time and completeness. Vital signs were recorded starting from 1 January 2000, laboratory data from 30 June 1999, anthropometric measurements from 10 January 2014, and medication prescription data from 10 May 2016. Diagnostic codes and physician notes were available from 10 December 2013 onwards.

Due to these differences in availability and recording practices, the number of patients included in each analysis (e.g., demographics, cardiometabolic risk factors, comorbidities) varied accordingly. These discrepancies are indicated in the results tables, and analyses were performed using all available data without imputation, consistent with the descriptive nature of this baseline cohort profile.

### Statistical analysis

The normality of the data was assessed using histograms, QQ plots, Shapiro-Wilk, or Kolmogorov-Smirnov tests. The results are presented as numbers and percentages for categorical variables, mean ± standard deviation (SD) for continuous variables with a normal distribution, or as median (interquartile range) for continuous variables with a skewed distribution. Descriptive statistics were computed using available-case analysis (row-wise deletion), without imputation for missing data. A sensitivity analysis was performed using data from 2016 onwards to determine if the sequential development of the UMMC EHR system affected the characteristics of the cohort (S1 Table). All analyses were performed using R version 4.4.0 (24 April 2024) – “Puppy Cup”.

### Ethics statement

This study received ethical approval from the UMMC Medical Research Ethics Committee (MREC) (MREC ID: 20191029−7950). As a retrospective observational study utilizing de-identified EHR with no direct patient interaction, individual patient consent was not required. All data were anonymized before analysis and securely stored on institutional servers, with access restricted to authorized researchers. The study adhered to institutional ethical guidelines governing the use of medical records for research while ensuring patient privacy and confidentiality.

### Patient and public involvement

No patients or members of the public were involved in the design, conduct, reporting, or dissemination plans of the present study.

## Results and discussion

### Cohort characteristics

The present MeLODY retrospective cohort comprised 15,702 adults with T2D (mean age at hospital visit 54.3 ± 12.6 years, mean diabetes duration 12.7 ± 4.8 years) (Table 1). The cohort was predominantly women (52.6%) and ethnically diverse, with 38.5% Malays, 33.0% Indians, and 27.5% Chinese. The mean HbA_1c_ was 8.9 ± 2.6%, with fewer than one-third having HbA_1c_ < 7%. The top three comorbidities were dyslipidaemia (87.1%), overweight/obesity (body mass index [BMI] ≥ 25 kg/m^2^; 69.4%), and hypertension (62.6%). Only 1 in 4 adults achieved a blood pressure target of < 130/80 mmHg or a low-density lipoprotein (LDL) cholesterol target of < 2.6 mmol/L. Nearly 80% of the cohort were treated with glucose-lowering medications, of whom 62.4% used metformin and one-third used either insulin or sulphonylureas. In terms of polypharmacy, nearly 40% of the cohort received ≥ 4 medications, whilst 1 in 5 adults were on ≥ 5 medications.

**Table 1:** Baseline characteristics of the retrospective MeLODY T2D cohort.

Characteristics	Number of patients	Results
**Demographics**		
Age at hospital visit, year	15,700	54.3 ± 12.6
Age at diabetes diagnosis, year	15,700	41.6 ± 9.5
Men, n (%)	15,702	7,450 (47.4%)
Ethnicity, n (%)		
Malay	15,465	5,957 (38.5%)
Indian	15,465	5,110 (33.0%)
Chinese	15,465	4,247 (27.5%)
Others	15,465	151 (1.0%)
**Cardiometabolic risk factors**		
Fasting plasma glucose, mmol/L	10,345	8.4 (6.1–12.9)
HbA_1c_ (NGSP, %)	6,931	8.9 ± 2.6
Systolic blood pressure, mmHg	2,454	140.2 ± 19.5
Diastolic blood pressure, mmHg	2,449	78.4 ± 10.1
Total cholesterol, mmol/L	7,971	5.4 ± 1.2
LDL-cholesterol, mmol/L	7,586	3.4 ± 1.1
Non-HDL cholesterol, mmol/L	7,833	4.3 ± 1.2
HDL-cholesterol, mmol/L	8,020	1.1 ± 0.3
Triglyceride, mmol/L	3,786	1.6 (1.2–2.3)
Body mass index, kg/m^2^	950	28.2 ± 6.2
eGFR, mL/min/1.73m^2^	11,389	98.6 ± 23.6
Age < 65 and FIB-4 ≥ 1.3	3,644	779 (21.4%)
Age ≥ 65 and FIB-4 ≥ 2.0	916	205 (22.4%)
**Comorbidities, n (%)**		
BMI ≥ 25 kg/m^2^	950	659 (69.4%)
BMI ≥ 30 kg/m^2^	950	302 (31.8%)
Dyslipidaemia	8,316	7,246 (87.1%)
Hypertension	2,598	1,627 (62.6%)
ASCVD	2,440	484 (19.8%)
Heart failure	2,440	118 (4.8%)
eGFR < 60mL/min/1.73m^2^	11,834	888 (7.5%)
Diabetic retinopathy	2,440	129 (5.3%)
Diabetes-related foot ulcer	2,440	183 (7.5%)
Lower-extremity amputation	2,440	105 (4.3%)
**Treatment targets attainment, n (%)**		
HbA_1c_ < 7% (53 mmol/mol)	6,931	1,926 (27.8%)
Blood pressure < 130/80 mmHg	2,449	607 (24.8%)
LDL-cholesterol < 1.4 mmol/L	7,586	200 (2.6%)
LDL-cholesterol < 1.8 mmol/L	7,586	533 (7.0%)
LDL-cholesterol < 2.6 mmol/L	7,586	1,862 (24.5%)
**Medications, n (%)**		
***Glucose-lowering***	1,573	1,218 (77.4%)
Insulin	1,573	515 (32.7%)
Metformin	1,573	981 (62.4%)
Sulphonylurea	1,573	516 (32.8%)
DPP4 inhibitors	1,573	107 (6.8%)
SGLT2 inhibitors	1,573	83 (5.3%)
GLP-1 RA	1,573	2 (0.1%)
***Blood pressure-lowering***	1,573	879 (55.9%)
Renin-angiotensin system inhibitors	1,573	577 (36.7%)
Beta-blockers	1,573	301 (19.1%)
Calcium channel blockers	1,573	477 (30.3%)
Diuretics	1,573	217 (13.8%)
Alpha-blockers	1,573	65 (4.1%)
***Lipid-lowering***	1,573	781 (49.7%)
Statins	1,573	775 (49.3%)
Fibrates	1,573	25 (1.6%)
Ezetimibe	1,573	8 (0.5%)
Anticoagulation	1,573	149 (9.5%)
Antiplatelet	1,573	375 (23.8%)
***Polypharmacy***		
≥ 4 medications	1,573	598 (38.0%)
≥ 5 medications	1,573	352 (22.4%)

Results are presented as mean ± standard deviation, median (interquartile range), or number (percentage). DPP4 inhibitors, dipeptidyl peptidase-4 inhibitors; eGFR, estimated glomerular filtration rate; FIB-4, fibrosis-4 index; GLP1-RA, glucagon-like peptide-1 receptor agonist; HbA_1c_, glycated haemoglobin; HDL, high-density lipoprotein; LDL, low-density lipoprotein; SGLT2 inhibitors, sodium-glucose cotransporter-2 inhibitors. The low sample size for medication data is due to the late introduction of the electronic prescription system in 2016.

### Findings to date

The present MeLODY retrospective cohort is distinctive in that it included data on adults with T2D who received outpatient care from a publicly funded academic institution, reflecting a population with greater complexity. It will allow trend analysis of outcomes of interest. Indeed, long-term follow-up of this cohort will yield valuable insights into incident clinical events, while also identifying gaps in care that could inform practice. We further compared the baseline data of the MeLODY cohort with other published T2D data from the region, including the 2022 TARGET-T2D study (which involved 5,094 adults from eight publicly funded specialist hospitals in Malaysia), the 2011–2020 Malaysian NDR (comprising 288,913 adults with T2D treated in publicly funded primary care clinics), as well as data from the 2013–2019 Singapore Health Service Diabetes Registry (SDR), and studies from Indonesia and Thailand [11,15–22].

#### Demographics.

Our cohort had a younger T2D population, with a mean age at hospital visit of 54.3 ± 12.6 years, compared to local and regional cohorts (TARGET-T2D: 59.0 ± 13.2 years, Malaysian NDR: 58.7 ± 10.8 years, SDR: 65.8 ± 13.7 years, Indonesia: 55.6 ± 9.8 years, and Thailand: 62.3 ± 11 years) [11,15,18,20,21]. Notably, the MeLODY cohort had a relatively longer diabetes duration of 12.7 ± 4.8 years, comparable to TARGET-T2D’s 14.8 ± 9.2 years, while other cohorts had a mean duration of 5–8 years. This suggests that Malaysia may have a higher burden of early-onset T2D, which tends to have an aggressive phenotype, compared to other ASEAN countries.

#### Treatment target attainment and guideline-directed medical therapy (GDMT).

The cardiometabolic risk factors in the present retrospective cohort highlighted several concerns compared to other ASEAN datasets. The mean HbA_1c_ in the present cohort was 8.9 ± 2.6%, which was higher than the SDR (7.2 ± 1.4%), Thailand (7.8 ± 2.0%), and Indonesia (8.0 ± 1.8%) [16,20,21]. The proportion of people achieving HbA_1c_ < 7% was 27.8% in the MeLODY cohort, comparable to 29.5% in the TARGET-T2D, but lower than the SDR (52.7%), and the Malaysian NDR (41%) [11,15,16,18]. This can be explained by the longer duration of diabetes and complex comorbidity profiles typical of a tertiary care cohort. Suboptimal health behaviours, limited access to care, therapeutic inertia, and treatment adherence can also contribute to the glycemic control observed.

Nearly 80% of the MeLODY cohort was prescribed glucose-lowering therapy, of whom one-third were using insulin, which surpassed the 20.2% as reported in the Malaysian NDR, indicating a higher burden of advanced diabetes in the former. The adoption of novel GDMT, such as sodium-glucose co-transporter 2 (SGLT2) inhibitors and glucagon-like peptide-1 receptor agonists (GLP-1 RA), was lower at 5.4% and 0.1%, respectively, in contrast to the TARGET-T2D cohort (37.8% and 5.3%). The difference in this context could be attributed to the distinct timeline, wherein most people in the MeLODY cohort were recruited before the expansion of their indications across the cardiovascular-kidney-metabolic conditions that happened in late 2021 [23,24]. Besides, there is often inertia for translating clinical trial evidence into routine practice, a challenge that is common at the global level, including in Malaysia [25]. The high cost of medications and country-specific reimbursement policies can also affect their uptake.

Every 1 in 4 adults achieved the blood pressure target of < 130/80 mmHg, which was comparable to the Malaysian NDR (24.3%) and the TARGET-T2D cohort (22.8%). A higher proportion was reported in the Thai and Singaporean cohorts at 29.8–39.1% and 36.0%, respectively [16,19,20]. Only 55.9% of the MeLODY cohort were prescribed blood pressure-lowering therapy, the lowest compared to other cohorts (Malaysian NDR 79.0%, Thailand 96.0%, and SDR 72.0%) [11,16,20]. Use of renin-angiotensin system inhibitors (36.7%) was also lower than in the Malaysian NDR (54.7%), TARGET-T2D (63.1%), and Thailand (54.9%) cohorts [11,15,17].

Lipid management is another area of concern. Nearly 90% of the MeLODY cohort had coexistent dyslipidaemia. However, similar to the blood pressure target, only 1 in 4 achieved the LDL-cholesterol target of < 2.6 mmol/L. Lipid-lowering therapy was prescribed to 49.7% of the cohort, which was lower than in the Malaysian NDR (71%), TARGET-T2D (90%), and SDR cohorts (79%). This shortfall may be additionally influenced by patient reluctance to take statins, driven by concerns over adverse effects, financial barriers, and general aversion to medication, with over 20% refusing treatment [26].

#### Comorbidities and complications.

The obesity prevalence of the present cohort, defined as BMI ≥ 30 kg/m^2^ (31.8%), was not directly comparable to other Malaysian and ASEAN cohorts. However, it was notably higher than its national average (21.8–22.4%) [8,27].

The prevalence of hypertension (62.6%) was lower than that in the NDR (81.6%), SDR (84.1%), and Thailand (78.1–79.9%) cohorts [11,17–19]. This difference can be attributed to our definition of hypertension used in this analysis, which relies on criteria such as the use of antihypertensive medications and/or blood pressure measurements (DBP ≥ 90 mmHg or SBP ≥ 140 mmHg) alone, rather than ICD-coded diagnosis.

The prevalence of atherosclerotic cardiovascular disease (ASCVD) in the MeLODY cohort was 19.8%, lower than the 29.8% reported in the TARGET-T2D cohort [15], with data unavailable for comparison in other cohorts. Limitations in the documenting and reporting of angina symptoms could contribute to the underestimation of ASCVD. The proportion of symptomatic heart failure stood at 4.9%, comparable to the 3.9% observed in the TARGET-T2D [15] and much higher than the national prevalence at 0.86% [28]. This aligns with the increased risk of heart failure in T2D, over twice that of the general population [29].

The MeLODY cohort recorded a 5.3% prevalence of diabetic retinopathy at baseline, which was lower than the Malaysian NDR (6.4%), Thailand (21.8%), and the SDR (11.6%) cohorts [11,17,22]. Globally, DR affects over 20% of people with T2D [30], with disparities likely due to a lack of periodic screening leading to delayed diagnosis.

Chronic kidney disease, defined as eGFR < 60 mL/min/1.73m^2^ in the present MeLODY cohort, was lower at 7.5%, compared to 29.8% as reported in the TARGET-T2D cohort [15]. The disparities were likely attributable to a lack of adherence to the guideline-recommended screening of microvascular complications with the use of urinary albumin:creatinine ratio due to its high cost and laboratory expertise [31,32].

The proportions of diabetes-related foot ulcer and lower-extremity amputation varied across different cohorts, with the MeLODY cohort showing a higher proportion at 7.5% and 4.3%, respectively, compared to the Malaysian NDR (0.4–0.9%) and the Singapore SDR (0.4–2.7%) cohorts [11,22]. These findings align with the global prevalence of diabetic foot ulcers at ~6% (rising to ~7% in hospital-based settings) [33] and a similar prevalence of lower-extremity amputation as reported in the 2006 Malaysian National Health and Morbidity Survey [34].

### Strengths and limitations

Some of the strengths of this cohort owe to its derivation from an EHR platform, capturing data on demographics, diagnosis, laboratory results, and medication use. This is especially noteworthy given its establishment in a middle-income country. Establishing such cohorts in LMICs is often hindered by financial and human resource limitations. The present MeLODY retrospective cohort includes a diverse representation of ethnicities, enhancing the generalizability of our findings to the broader Malaysian and ASEAN populations. Spanning from 2002 to 2020, the present cohort can facilitate longitudinal analyses. As a publicly funded academic institution providing extensive subspecialty services, the present cohort also predominantly includes people with T2D who are at increased risk of multiple comorbidities such as ASCVD, heart failure, CKD, and cancer that are less commonly encountered in primary and secondary care settings.

Several limitations should be acknowledged. Firstly, the reliance on electronic health records introduces variability dependent on physicians’ adherence to comprehensive and accurate diagnosis, and history documentation. Secondly, Table 1 shows the variability in data completeness, with some variables, such as anthropometric measurements and medications, having lower sample sizes than other variables. This is because the electronic prescribing system was initiated much later in 2016. However, the sensitivity analysis with data captured from 2016 reported mostly minor differences when compared with the overall cohort (S1 Table). Lastly, there is a scarcity of detailed information on socioeconomic status, health behaviour (smoking, alcohol, physical activity, and diet), and psychosocial assessment.

## Conclusions

The present MeLODY retrospective cohort revealed that people with T2D were not optimally managed, with notable gaps in achieving key treatment targets and outcomes. This highlights the urgent need for (1) linkages with other disease and death registries to provide a more comprehensive understanding of clinical outcomes and data robustness, and (2) a well-designed prospective cohort study to enable the collection of high-quality and standardized data, providing valuable benchmarking insights to guide monitoring and treatment. By identifying these actionable gaps and setting achievable goals, this approach could pave the way for better health outcomes and resource allocations.

## Supporting information

S1 Table Baseline characteristics of the retrospective MeLODY T2D cohort with entry dates > 2016. Results are presented as mean ± standard deviation, median (interquartile range), or number (percentage). DPP4 inhibitors, dipeptidyl peptidase-4 inhibitors; eGFR, estimated glomerular filtration rate; FIB-4, fibrosis-4 index; GLP1-RA, glucagon-like peptide-1 receptor agonist; HbA_1c_, glycated haemoglobin; HDL, high-density lipoprotein; LDL, low-density lipoprotein; SGLT2 inhibitors, sodium-glucose cotransporter-2 inhibitors.
(DOCX)

S1 Text Machine learning model summary. (DOCX)
