# Supplementary material for: Cohort profile: The Multiethnic Lifestyle, Obesity and Diabetes Registry in Malaysia (MeLODY) retrospective cohort in a middle-income country in Southeast Asia
Source: PLoS One. 2025 Sep 9;20(9):e0331571. doi: 10.1371/journal.pone.0331571 (PMC12419649; doi:10.1371/journal.pone.0331571)
Supplement: S1 Table — Results are presented as mean ± standard deviation, median (interquartile range), or number (percentage). DPP4 inhibitors, dipeptidyl peptidase-4 inhibitors; eGFR, estimated glomerular filtration rate; FIB-4, fibrosis-4 index; GLP1-RA, glucagon-like peptide-1 receptor agonist; HbA1c, glycated haemoglobin; HDL, high-density lipoprotein; LDL, low-density lipoprotein; SGLT2 inhibitors, sodium-glucose cotransporter-2 inhibitors. (DOCX) [file pone.0331571.s001.docx]

| **Characteristics** | **Number of patients** | **Results** |
| --- | --- | --- |
| **Demographics** |  |  |
| Age at hospital visit, year | 1,573 | 56.6 ± 13.2 |
| Age at diabetes diagnosis, year | 1,573 | 43.3 ± 9.8 |
| Men, n (%) | 1,573 | 7,450 (47.4%) |
| Ethnicity, n (%) |  |  |
| Malay | 1,516 | 585 (38.6%) |
| Indian | 1,516 | 523 (34.5%) |
| Chinese | 1,516 | 368 (24.3%) |
| Others | 1,516 | 40 (2.6%) |
| **Cardiometabolic risk factors** |  |  |
| Fasting plasma glucose, mmol/L | 1,060 | 9.6 (6.6 - 15.2) |
| HbA_1c_ (NGSP, %) | 1,120 | 9.3 ± 2.6 |
| Systolic blood pressure, mmHg | 1,501 | 140.4±18.9 |
| Diastolic blood pressure, mmHg | 1,499 | 78.0±9.8 |
| Total cholesterol, mmol/L | 855 | 5.1±1.3 |
| LDL-cholesterol, mmol/L | 847 | 3.0±1.1 |
| Non-HDL cholesterol, mmol/L | 855 | 4.0±1.2 |
| HDL-cholesterol, mmol/L | 880 | 1.2±0.3 |
| Triglyceride, mmol/L | 881 | 1.6 (1.2-2.3) |
| Body mass index, kg/m^2^ | 552 | 28.0±6.4 |
| eGFR, mL/min/1.73m^2^ | 1,312 | 99.1±29.5 |
| Age < 65 and FIB-4 ≥ 1.3 | 389 | 109 (28.0%) |
| Age ≥ 65 and FIB-4 ≥ 2.0 | 156 | 49 (31.4%) |
| **Comorbidities, n (%)** |  |  |
| BMI ≥ 25 kg/m^2^ | 552 | 372 (67.4%) |
| BMI ≥ 30 kg/m^2^ | 552 | 164 (29.7%) |
| Dyslipidaemia | 1,527 | 1,030 (67.5%) |
| Hypertension | 1,532 | 1,100 (71.8%) |
| ASCVD | 1,483 | 352 (23.7%) |
| Heart failure | 1,483 | 88 (5.9%) |
| eGFR < 60mL/min/1.73m^2^ | 1,312 | 150 (11.4%) |
| Diabetic retinopathy | 1,483 | 103 (6.9%) |
| Diabetes-related foot ulcer | 1,483 | 108 (7.3%) |
| Lower-extremity amputation | 1,483 | 73 (4.9%) |
| **Treatment targets attainment, n (%)** |  |  |
| HbA_1c_ < 7% (53 mmol/mol) | 1,120 | 265 (23.7%) |
| Blood pressure < 130/80 mmHg | 1,499 | 362 (24.1%) |
| LDL-cholesterol < 1.4 mmol/L | 847 | 39 (4.6%) |
| LDL-cholesterol < 1.8 mmol/L | 847 | 108 (12.8%) |
| LDL-cholesterol < 2.6 mmol/L | 847 | 314 (37.1%) |
| **Medications, n (%)** |  |  |
| ***Glucose-lowering*** | 1,573 | 1,218 (77.4%) |
| Insulin | 1,573 | 515 (32.7%) |
| Metformin | 1,573 | 981 (62.4%) |
| Sulphonylurea | 1,573 | 516 (32.8%) |
| DPP4 inhibitors | 1,573 | 107 (6.8%) |
| SGLT2 inhibitors | 1,573 | 83 (5.3%) |
| GLP-1 RA | 1,573 | 2 (0.1%) |
| ***Blood pressure-lowering*** | 1,573 | 879 (55.9%) |
| Renin-angiotensin system inhibitors | 1,573 | 577 (36.7%) |
| Beta-blockers | 1,573 | 301 (19.1%) |
| Calcium channel blockers | 1,573 | 477 (30.3%) |
| Diuretics | 1,573 | 217 (13.8%) |
| Alpha-blockers | 1,573 | 65 (4.1%) |
| ***Lipid-lowering*** | 1,573 | 781 (49.7%) |
| Statins | 1,573 | 775 (49.3%) |
| Fibrates | 1,573 | 25 (1.6%) |
| Ezetimibe | 1,573 | 8 (0.5%) |
| Anticoagulation | 1,573 | 149 (9.5%) |
| Antiplatelet | 1,573 | 375 (23.8%) |
| ***Polypharmacy*** |  |  |
| ≥ 4 medications | 1,573 | 598 (38.0%) |
| ≥ 5 medications | 1,573 | 352 (22.4%) |
