# Supplementary material for: Cohort profile: The Multiethnic Lifestyle, Obesity and Diabetes Registry in Malaysia (MeLODY) retrospective cohort in a middle-income country in Southeast Asia
Source: PLoS One. 2025 Sep 9;20(9):e0331571. doi: 10.1371/journal.pone.0331571 (PMC12419649; doi:10.1371/journal.pone.0331571)
Supplement: S1 Text — (DOCX) [file pone.0331571.s002.docx]

**S1 Text. Machine learning model summary.**

In the electronic health records, the date of diabetes diagnosis and therefore, the duration of diabetes, was not consistently or comprehensively documented. Consequently, a machine learning model was trained on a cohort with a high degree of similarity, comprising tertiary healthcare centres in the Klang Valley region [15]. The dataset was split into training (70%) and testing (30%) subsets, and 5-fold cross-validation was used to tune hyperparameters where applicable. The target variable was the estimated number of years since diabetes onset, modelled as a continuous outcome.

An ensemble model was constructed using the SuperLearner package in R software, incorporating predictions from linear regression, LASSO regression (glmnet), regression trees (rpart), and XGBoost (xgboost). Predictor selection was guided by variable importance rankings, derived from a combination of analyses using regression trees, LASSO regression, and linear regression methods. Key features included age at cohort entry, insulin use, HbA_1c_, estimated glomerular filtration rate (eGFR), comorbidities (e.g., hypertension, coronary artery disease, end-stage kidney disease), anthropometric measures (weight, height, and calculated body mass index), and lipid parameters.

The ensemble model outperformed all individual models on the test set. Predictive performance is summarized below:

| **Model** | | **RMSE** | **R^2^** | **MAE** |
| --- | --- | --- | --- | --- |
| Naïve mean prediction (baseline) | | 0.988 | 0.000 | 0.793 |
| Linear Regression | | 0.803 | 0.340 | 0.620 |
| LASSO Linear Regression | | 0.813 | 0.332 | 0.633 |
| Regression Tree | 0.830 | | 0.298 | 0.644 |
| XGBoost | 0.801 | | 0.344 | 0.616 |
| SuperLearner (ensemble model) | 0.795 | | 0.356 | 0.614 |

The ensemble model was thus retained as the final prediction model, given its superior accuracy and generalization performance across all metrics.
